# A stool based qPCR for the diagnosis of TB in children and people living with HIV in Uganda, Eswatini and Mozambique (Stool4TB): a protocol for a multicenter diagnostic evaluation

**DOI:** 10.1186/s12879-023-08708-9

**Published:** 2024-02-21

**Authors:** Lucia Carratala-Castro, Willy Ssengooba, Alex Kay, Sozinho Acácio, Joanna Ehrlich, Andrew R DiNardo, Nosisa Shiba, Joachim K Nsubuga, Shilzia Munguambe, Belén Saavedra-Cervera, Patricia Manjate, Durbbin Mulengwa, Busizwe Sibandze, Mangaliso Ziyane, George Kasule, Edson Mambuque, Moorine Penninah Sekadde, Eric Wobudeya, Moses L Joloba, Jan Heyckendorf, Christoph Lange, Sabine Hermans, Anna Mandalakas, Alberto L. García-Basteiro, Elisa Lopez-Varela, on behalf of Stool4TB Global Partnership, Sergi Sanz, Makhosazana Dlamini, Gcinile Dlamini, Nomathemba Dlamini, Nkulungwane Mthethwa, Nokwanda Kota, Mbongeni Dube, Nontobeko Maphalala, Babongile Nkala, Faith Dlamini, Fortunate Shabalala, Sindisiwe Dlamini, Gugu Maphalala, Lindiwe Dlamini, Sisi Dube, Lee Joao Fonseca, Nércio Machele, Miguel Cumbe, Agostinho Lima, Katia Magul, Gustavo Tembe, Benilde Violeta Mudumane, Farida Cebola, Jorcelina Rungo, Alberto Bila Junior, Neide Gomes, Patricia Mwachan, Maria Nassolo, Sujan Katuwal, Matthew Ang, Anca Vasiliu, Rojelio Mejía, Jason Bacha, Debrah Vambe, Abigail Seeger, Irina Kontsevaya, Collins Musia, Lilian Komba, Lwijisyo Minga, Lumumba Mwita, Mtafya Bariki, Nyanda Elias Ntinginya

**Affiliations:** 1 Centro de Investigação em Saúde de Manhiça (CISM), Mozambique Maputo,; 2 Fundación Privada Instituto de Salud Global Barcelona (ISGlobal), Spain Barcelona,; 3 Makerere University, Kampala, Uganda; 4 Baylor College of Medicine (BCM), Houston, TX USA; 5 Baylor College of Medicine -Children’s Foundation Eswatini, Mbabane, Eswatini Swaziland; 6 Instituto Nacional de Saúde (INS), Ministério da Saúde de Moçambique, Mozambique Maputo,; 7 Radboud UMC, Nijmegen, Netherlands; 8 Amsterdam UMC, location University of Amsterdam, Department of Global Health, Amsterdam Institute for Global Health and Development, Amsterdam, the Netherlands; 9 National Tuberculosis Reference Laboratory, Mbabane, Eswatini Swaziland; 10 National Tuberculosis and Leprosy Program, Uganda Kampala,; 11 Department of Internal Medicine I, University Medical Center Schleswig-Holstein, Kiel, Germany; 12 Division of Clinical Infectious Diseases, Research Center Borstel, Borstel, Germany; 13 German Center for Infection Research (DZIF), Partner Site Hamburg-Lübeck-Borstel-Riems, Borstel, Germany; 14 Respiratory Medicine and International Health, University of Lübeck, Lübeck, Germany; 15 Baylor College of Medicine and Texas Children Hospital, Global TB Program, Houston, TX USA; 16 Amsterdam UMC, location University of Amsterdam, Centre for Tropical Medicine and Travel Medicine, Department of Infectious Diseases, Amsterdam, the Netherlands; 17 Research Center Borstel, Borstel, Germany; 18 Barcelona Institute for Global Health, Barcelona, Spain; 19 Baylor Children’s Foundation Eswatini, Mbabane, Swaziland; 20 University of Eswatini, Kwaluseni, Eswatini; 21 Eswatini Health Laboratory Services, Ezulwini, Eswatini; 22 Eswatini National Tuberculosis Control Program, Manzini, Eswatini; 23 Centro de investigação de Saúde de Manhiça, Mozambique Manhiça Maputo,; 24 Makerere University, Uganda Kampala,; 25 Baylor College of Medicine Houston, Houston, USA; 26 Research Center Borstel, Sülfeld, Germany; 27 Baylor Children’s Foundation Tanzania, Mwanza, Tanzania; 28 Mbeya Medical Research Center, Mbeya, Tanzania

**Keywords:** Tuberculosis, Children, PLHIV, Stool, Molecular diagnostics

## Abstract

**Background:**

Tuberculosis (TB) is a major cause of mortality worldwide. Children and people living with HIV (PLHIV) have an increased risk of mortality, particularly in the absence of rapid diagnosis. The main challenges of diagnosing TB in these populations are due to the unspecific and paucibacillary disease presentation and the difficulty of obtaining respiratory samples. Thus, novel diagnostic strategies, based on non-respiratory specimens could improve clinical decision making and TB outcomes in high burden TB settings. We propose a multi-country, prospective diagnostic evaluation study with a nested longitudinal cohort evaluation to assess the performance of a new stool-based qPCR, developed by researchers at Baylor College of Medicine (Houston, Texas, USA) for TB bacteriological confirmation with promising results in pilot studies.

**Methods:**

The study will take place in high TB/HIV burden countries (Mozambique, Eswatini and Uganda) where we will enroll, over a period of 30 months, 650 PLHIV (> 15) and 1295 children under 8 years of age (irrespective of HIV status) presenting pressumptive TB. At baseline, all participants will provide clinical history, complete a physical assessment, and undergo thoracic chest X-ray imaging. To obtain bacteriological confirmation, participants will provide respiratory samples (1 for adults, 2 in children) and 1 stool sample for Xpert Ultra MTB/RIF (Cepheid, Sunnyvale, CA, USA). *Mycobacterium tuberculosis* (*M.tb*) liquid culture will only be performed in respiratory samples and lateral flow lipoarabinomannan (LF-LAM) in urine following WHO recommendations. Participants will complete 2 months follow-up if they are not diagnosed with TB, and 6 months if they are. For analytical purposes, the participants in the pediatric cohort will be classified into “confirmed tuberculosis”, “unconfirmed tuberculosis” and “unlikely tuberculosis”. Participants of the adult cohort will be classified as “bacteriologically confirmed TB”, “clinically diagnosed TB” or “not TB”. We will assess accuracy of the novel qPCR test compared to bacteriological confirmation and Tb diagnosis irrespective of laboratory results. Longitudinal qPCR results will be analyzed to assess its use as treatment response monitoring.

**Discussion:**

The proposed stool-based qPCR is an innovation because both the strategy of using a non-sputum based sample and a technique specially designed to detect M.tb DNA in stool.

**Protocol registration details:**

ClinicalTrials.gov Identifier: NCT05047315.

## Background

Tuberculosis (TB) remains a major cause of morbidity and mortality worldwide, resulting in 10.6 million incident cases and 1.6 million deaths in 2021 [1]. The African region is one of the most TB-affected areas in the world, representing approximately 23% of the global TB disease burden, 26% of deaths among HIV negative people and 73% of the deaths among people living with HIV (PLHIV) [2]. Both children and PLHIV have an increased risk of TB progression and mortality, particularly in the absence of rapid diagnosis [1, 3]. Recent modeling studies suggest that around 96% of children dying from TB do not receive adequate treatment and from those under 5 years of age 80% are not even diagnosed [4]. Early and appropriate TB diagnosis in these two populations is a key pillar of the WHO End TB Strategy, where specific emphasis is placed on the discovery, development, and rapid uptake of new diagnostic tools and effective strategies for their implementation and scale-up [5].

Despite some advances in TB diagnostics in recent years, such as the emergence of Xpert Ultra and LF-LAM, diagnosing and confirming TB remains a challenge particularly among children and immunocompromised PLHIV. In these groups, TB often presents without traditional TB clinical symptoms, [6, 7] making passive case detection based on symptom screening less effective at detecting TB. The paucibacillary nature of disease [8] in children and PLHIV, and challenges in obtaining respiratory samples due to difficulty spontaneously expectorating sputum also create obstacles towards bacteriological confirmation. Thus, new diagnostic strategies, based on the use of easy-to-collect non-invasive specimens, could improve clinical decision making and TB outcomes TB high burden settings.

Since children and PLHIV are less likely to produce quality sputum, increased interest has been placed on exploring new specimens for TB diagnosis and sputum free diagnostics was listed as one of the WHO’s high priority target products for TB diagnostics [9]. Recent studies have identified stool as an effective child-friendly specimen, since airway secretions are normally cleared into the digestive system. These studies went on to inform the most recent WHO guidelines, which now recommend stool as an initial sample for microbiological diagnosis in children [10].

Despite its increasing availability in high TB burden settings, the 4^th^ generation Xpert® Ultra in sputum (Cepheid Inc., Sunnyvale, CA, USA) confirms TB in 90% of adult cases with HIV-infection[11] and 62–89% of culture-positive pediatric cases [12]. While mycobacterial culture is the accepted reference standard for assessing novel TB diagnostic tests, it only confirms TB in 10–50% of children clinically diagnosed with pulmonary TB [13–16] and about half of PLHIV starting on anti-tuberculosis treatment (ATT).

To address this diagnostic gap, researchers at Baylor College of Medicine (Houston, Texas, USA) developed a novel, stool bead-based, real-time quantitative PCR (qPCR) diagnostic test for TB. A pilot study evaluating this qPCR have shown its ability to increase TB case confirmation among adults in Eswatini and children in Tanzania with clinically diagnosed, bacteriologically negative TB (by sputum Xpert) by 12% and 19%, respectively. This study also points to the test’s ability to predict TB treatment failure [17]. In Eswatini, the detection of *M.tb* DNA in the stool after 2 months of ATT was associated with treatment failure, death, or DR-TB (RR 3.5, 95% CI: 1.32- 9.64; *p* = 0.015) [17]. Thus, this qPCR platform provides an important opportunity to explore the utility of a qPCR for treatment monitoring via quantification of *M.tb* burden in stool. In addition, this test could be combined with novel approaches to assess *M. tb* viability using molecular mycobacterial load assays [18].

Stool Xpert MTB/RIF was reported to have a limit of detection (LOD) greater than 267 CFU/mL [19]. In contrast, when using a bead-based DNA isolation kit designed for soil (MP Fast DNA, MPBio), the LOD was lowered to 96 Mtb Colony Forming Units (CFU) per 50 mg of stool (95% CI 84–105 CFU), which approximately represents 5 CFU/mL [20, 21]. There is still little evidence about the performance of Xpert Ultra on stool, but a recent Cochrane review reports a sensitivity of around 60% against culture (varying from 39 to 100% including data from unpublished cohorts) [12]. For Xpert Ultra the LOD has been lowered to 16 CFU/mL. This shows some room for improvement, for which we expect the qPCR, combined with the DNA isolation kit developed for stool specimens, to present with a higher sensitivity than Xpert® Ultra. Therefore, we propose a diagnostic evaluation to assess the performance of this stool-based qPCR for TB bacteriological confirmation among PLHIV and children with the hypothesis that it may present an increased sensitivity for TB diagnosis compared to currently recommended microbiological tests.

## Methods

### Aim and objectives

The primary objective of the Stool4TB study is to evaluate the diagnostic performance of the stool bead-based real-time quantitative PCR (qPCR) platform for TB diagnosis in children < 8 years and PLHIV compared to a composite reference standard that includes sputum (induced sputum and/or gastric aspirate in children) and stool Xpert Ultra, sputum (induced sputum and/or gastric aspirate in children) culture, and urine LF-LAM (among people living with HIV).

As a secondary objective, we will compare the diagnostic accuracy of the novel stool qPCR assay to the other tests against clinical diagnosis and a bacteriological reference standard [22]. We will also evaluate the quantitative stool qPCR platform as a tool to monitor treatment response in these two populations. Samples collected from these cohorts will be used to develop a biorepository of well characterized samples to enable future TB related research as an exploratory objective.

### Study design

This is a multi-national, prospective, diagnostic evaluation study with a nested longitudinal cohort evaluation. The total duration of the study will be 60 months, with a 30-month recruitment period in which different samples will be collected at baseline to assess the diagnostic performance of the qPCR assay. Recruitment in adults began November 2021 and will end by May 2024. In children recruitment began in April 2022 and will end by September 2024. All participants initiating TB treatment will be followed until treatment completion in order to assess treatment outcomes and the potential of the qPCR assay to be used as a treatment monitoring tool. Those participants not initiating TB treatment will be followed for 2 months after recruitment to accurately rule out TB diagnosis.

### Study settings

The study will be performed in three high TB/HIV burden settings: [23–32] the Manhiça Health Research Centre (CISM) located in the Manhiça District of Maputo Province in southern Mozambique; Baylor College of Medicine Children’s Foundation-Eswatini Clinical Centre of Excellence (COE) in Mbabane, Eswatini; and Makerere University College of Health Sciences (MaKCHS) in Kampala, Uganda. In Mozambique, entry points include the District Hospital of Manhiça (MDH) facility which supports the 10 primary health centres that provide referral services to the MDH. In Eswatini, entry points include the Mbabane Government Hospital (MGH), Raleigh Fitkin Memorial Hospital, Pigg’s Peak Hospital, Dvokolwako Health Center, and Baylor Clinical Centre of Excellence (COE). In Uganda, recruitment will take place at the largest TB care clinic, the Pediatric TB ward and the TB ward 5 and 6 all situated at Mulago National Referral and teaching Hospital (MNRH), as well as other high-volume TB/HIV clinics within Kampala Capital City Authority (KCCA) in collaboration with the National TB and Leprosy program (NTLP).

### Study population

Participants ≥ 15 years of age living with HIV and children less than 8 years of age (irrespective of HIV status) presenting with signs and symptoms of presumptive TB to the entry points of the selected sites will be referred to the study team. Individuals with presumptive TB or their guardians if they are under 18 years old will be invited to complete informed consent and participate in the study after screening has ensured that they meet the study inclusion criteria. Participants from 15 to 17 years old will be also required to assent in order to enter the study.

Participants will be eligible for the adult cohort based on the following inclusion criteria: a) 15 or more years of age, b) confirmed HIV infection (antibody-based or molecular test), and c) reporting any of the following symptoms: cough, fever, night sweats or unintentional weight loss (any duration).

**Fig. 1:**
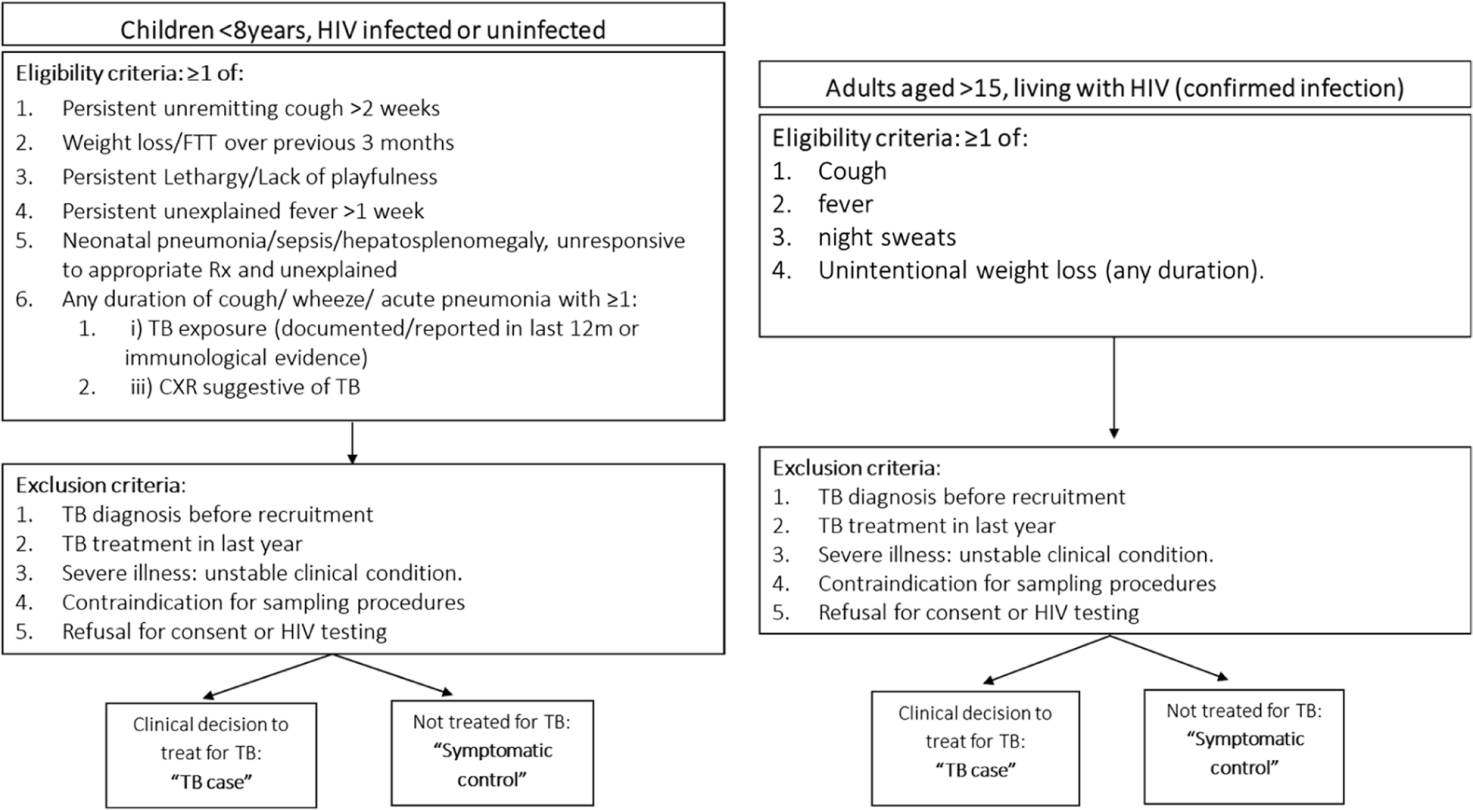
Inclusion/Exclusion criteria

Children will be included in the study if: a) age under 8 years of age irrespective of HIV status, and b) reporting any of the following symptoms: 1) Persistent unremitting cough (or cough significantly worse than usual in child with chronic lung disease including HIV-related) of > 2 weeks duration, unresponsive to a course of appropriate antibiotics (when clinically indicated); 2) Poor growth documented over the preceding 3 months [clear deviation from a previous growth trajectory and/or documented crossing of centile lines in the preceding 3 months and/or; weight-for-age, or weight-for-height Z-score of ≤ 2 in the absence of information on previous/recent growth trajectory AND not responding to nutritional rehabilitation (or to antiretroviral therapy if HIV-infected)]. 3) Persistent unexplained lethargy or reduced playfulness/activity reported by the caregiver. 4) Persistent (> 1w) unexplained fever (> 38C), reported by a guardian or objectively recorded at least once. 5) In infants 0–60 days, also: unresponsive neonatal pneumonia or unexplained hepatosplenomegaly OR sepsis-like illness (all other more common causes excluded and/or not responding to appropriate therapy/ broad-spectrum antibiotics/antivirals) [33] (see Fig. 1).

Participants will be excluded from both study groups if they have received a TB diagnosis before recruitment, have received TB treatment in the last year, do not provide consent (or assent if applicable) for study participation or HIV testing when status is unknown, have severe illness resulting in an unstable condition or if there is an absolute contra-indication to any of sampling procedures required by the study (ie: acute severe asthma, pertussis syndrome etc.) (see Fig. 1).

### Study visits, assessments and clinical procedures

After written informed consent has been obtained from the adult participants or the children’s guardians, the study clinician or nurse will perform a full anamnesis, complete the clinical evaluation, obtain a chest X-ray (AP/PA and lateral for children) and conduct sampling for bacteriological confirmation. For children, 2 respiratory specimens (either induced sputum, gastric aspirate or nasopharyngeal aspirate), 1 urine sample (for those living with HIV) and 1 stool sample will be collected as soon as possible. In the adult cohort, only one sputum sample will be required. Blood samples will be collected if HIV test has not been performed in the last 6 months in children, and if viral load and CD4 count has not been performed in those PLHIV in the previous 3–6 months, and TST/IGRA will be performed at baseline, if available (see Table 1 for Schedule of events, SoE).

Those participants not starting ATT will be referred to routine services for clinical management and a study clinical follow-up visit will be conducted at 2 weeks and 2 months to ascertain the TB free phenotype. If the participant is still symptomatic by two months of follow-up, full resampling and clinical assessment will be done as detailed in the schedule of events (Table 1). Parent(s)/guardian(s) are invited to bring their child back to the hospital in case of new symptoms for an unscheduled (extra) visit. The decision to start ATT will be made by either healthcare system doctors or study clinicians in accordance with the local guidelines at each of the sites.

**Table 1:** Schedule of events

	**Baseline**	**Week 2**	**Month 2**	**Months 4**	**Month 6**
**PRESUMPTIVE TB**					
Past medical history	X				
TB exposure assessment^a^	X		X		
Full clinical assessment	X	X (tel adults)^b^	X		
CD4, HIV viral load (if HIV positive)^c^	X				
Chest X-Ray	X				
Diagnostic Samples	X		if symptoms		
SPUTUM (ultra, culture, Biorepository)					
STOOL (ultra, qPCR, Bio)					
URINE (LAM, bio)					
Blood^d^	IC (60)				
**TB CASE**^**e**^					
Clinical assessment	X	X	X	X	X
Chest X-Ray					X
ATT response (sputum, stool)		cTB	cTB	cTB	cTB
Blood^d^	cTB (200)	cTB (200)		cTB (200)	cTB (200)

*IC* Ill controls, *cTB* confirmed TB

^a^ TB exposure assessment will be performed via questionnaire in every participant and TST/IGRA will be performed at baseline if available on site

^b^On week 2, the clinical assessment will be performed by telephone in adults without symptoms

^c^If HIV infected and have not had VL and CD4 count in the last 3 months. Up to six months VL and CD4 counts will be accepted

^d^Blood (PAXgene, QF, serum): QuantiFERON will be collected in baseline and four months for those starting treatment and at baseline in 60 ill controls (10 pressumptive TB participants that do not meet the criteria for TB disease per category and site). PAXgene and serum will be collected at baseline, week 2, month 4 and month 6 for those starting treatment, and at baseline in 60 healthy controls

^e^If a decision is made to start ATT after the baseline visit, the schedule of follow-up visits should be adapted so that the TB treatment start date will be the new “baseline” date

For those starting ATT, follow-up will be conducted until the end of treatment with visits at 2 weeks, 2 months, 4 months and 6 months after ATT initiation (and an extra visit at the end of treatment if the treatment lasts more than 6 months). Among people who have bacteriologically confirmed TB, one respiratory sample, one stool sample and a urine sample will be collected at each follow-up visit until negative bacteriological samples have been obtained for at least two consecutive visits. Chest X-ray will be performed at the end of ATT.

Blood will be collected in those bacteriologically confirmed at the start of ATT, week 2 post ATT, month 4 post ATT and at the end of treatment to conduct treatment monitoring mRNA signatures. After study sampling is completed according to the schedule of events (Table 1), remaining samples will be stored in a biorepository for use in future exploratory TB research.

### Laboratory procedures

All respiratory samples including gastric aspirate (diagnostic or treatment monitoring samples) will undergo Xpert Ultra and Mycobacterium Growth Indicator Tubes (MGIT) liquid medium and incubated in the Bactec MGIT 960 mycobacterial detection instrument (Becton Dickinson Microbiology System, BD, USA) and, if available, solid culture (comparator tests). All assays will be performed within 24 h of collection at the reference laboratories (CISM, MU and Eswatini BSL 3.

Results will be interpreted following manufacturer. Following the molecular assays, cultures will be performed. Raw samples will be decontaminated by applying the Kubica method [34]. From the decontaminated pellet, 500 µl will be inoculated into liquid medium* (Mycobacterium Growth Indicator Tubes*—MGIT) and incubated in the Bactec MGIT 960 mycobacterial detection instrument (Becton Dickinson Microbiology System, BD, USA), and 200 µl will be inoculated into solid media (Lowenstein_Jensen—LJ). After 42 days without growth (for MGIT), or 8 weeks (for LJ), cultures will be classified as negative.

All stool samples (for diagnosis or treatment monitoring) will undergo qPCR and Xpert Ultra testing. DNA will be extracted from 50 mg of stool using the MP FastDNA for Soil Kit (MP Biochemicals) in preparation for the stool qPCR. In brief, ethanol will be removed and 50 mg of stool are homogenized using bead-beating with 100μL of DNA eluted by the MPFast DNA soil kit. *Mtb*-specific primers and black hole quencher FAM-labeled minor groove binder probes have been selected from previously described *M. tb*-specific IS6110 sequences [35]. Of note, our qPCR incorporates a control for extraction/PCR inhibition. Stool will be aliquoted, frozen at -80 °C and run in batches at study-specific labs in Mbabane (Baylor COE), Manhica (CISM), and Makerere University (MU). Xpert Ultra will be performed following the KNCV simple one-step (SOS) method [36]. Results will be interpreted following manufacturer guidelines.

Lateral flow urine lipoarabinomannan assays (LF-LAM) from Abbott® will be performed in participants with presumptive TB and confirmed HIV infection in line with the most recent WHO guidelines [37]. The LF-LAM assay will be performed following manufacturer’s instructions. Urine samples will be preserved in -20ºC for future LF-LAM testing if the test is not immediately available. The remaining urine will be stored at -80º for the biorepository.

### Endpoints

#### Definitions

For analysis, the participants in the pediatric cohort will be classified as ‘confirmed tuberculosis’, ‘unconfirmed tuberculosis’ and ‘unlikely tuberculosis’ according international consensus pediatric endpoint definitions [22].  “TB case” definition will include both ‘confirmed’ and ‘unconfirmed’ tuberculosis. “Unlikely TB” will be used as reference for specificity (see Table 2).

**Table 2:** Definitions for the pediatric cohort

**TB case**	**“Confirmed tuberculosis”**	*M. tb* to be laboratory-confirmed (culture or NAAT-based assay) from at least 1 specimen or positive urine LAM
	**“Unconfirmed tuberculosis”**	Bacteriological confirmation not obtained and at least 2 of the following: 1. symptoms/signs suggestive of tuberculosis, 2. chest radiograph consistent with tuberculosis, 3. close tuberculosis exposure or immunologic evidence of *M. tuberculosis* infection 4. positive response to tuberculosis treatment (requires documented positive clinical response on tuberculosis treatment—no time duration specified)
**Not TB case**	**“Unlikely tuberculosis”**	bacteriological confirmation not obtained and criteria for “unconfirmed tuberculosis” not met

For the adult cohort participants will be classified as “bacteriologically confirmed TB” or “clinically diagnosed TB”. “TB case” definition will be equivalent to decision to start treatment irrespective of bacteriological confirmation so it will include both “bacteriologically confirmed TB” and “clinically diagnosed TB”

#### Primary endpoint

Sensitivity and other test parameters, such as specificity, positive predictive value (PPV) and negative predictive value (NPV), of the qPCR test will be calculated with respect to a composite reference standard including any of the other tests (induced sputum and gastric aspirate Xpert Ultra and MGIT culture, stool Ultra or TB-LAM in HIV-positive individuals).

#### Secondary endpoints

Sensitivity and other parameters (specificity, PPV and NPV) of the qPCR test with respect to the “TB case” definition.

Accuracy of stool qPCR and each of the other microbiological confirmation tests separately against the “TB case” definition.

Treatment response by monitoring time to negativity of the qPCR on stool after treatment initiation and comparing with standard mechanisms for treatment monitoring (symptom resolution and culture negativity on sputum) as well as predicting outcome.

### Data synthesis and analysis

We will summarize demographic characteristics of the samples and will perform a descriptive analysis of endpoints and other relevant clinical variables. Quantitative variables will be summarized using means, standard deviation, median and interquartile range. Categorical variables will be analysed using frequencies and proportions. We will analyze the mechanisms that contribute to missing data, which will guide subsequent imputation methods to address missingness. An accuracy analysis will be performed for both study groups (children and PLHIV) with bacteriologically confirmed tuberculosis as a reference standard and qPCR assay on stool as the index test under evaluation. We will calculate sensitivity *(primary endpoint)* and other test parameters: specificity, PPV and NPV, of the qPCR test compared to composite reference standard that includes any of the other tests positive (including induced sputum and gastric aspirate Xpert Ultra and MGIT culture, stool Ultra or LF-LAM). We will also calculate sensitivity (and other parameters: specificity, PPV and NPV) of the qPCR test *(secondary endpoint)* with respect to a clinical case definition. Accuracy will also be assessed for stool qPCR and each of the other microbiological confirmation tests separately against the clinical case definition (*secondary endpoint)*, in order to compare the performance of each test.

### Power and sample size

Sample size calculations were based on the primary endpoint to compare the qPCR assay performance to the bacteriological composite reference standard. Calculations were performed based on the following assumptions: (1) A proportion of laboratory confirmed TB of 8.5% in the pediatric cohort and 20% in the adult cohort. (2) 80% sensitivity of qPCR against the composite reference standard with a precision of 7.5% around the estimate in the child cohort and 7% in the adult cohort. According to these assumptions, the required sample size for the cohort is 1295 children (525 in Mozambique and Uganda and 245 in Eswatini), and 650 adults (250 in Eswatini and Mozambique and 150 in Uganda).

### Biorepository

Baseline samples, including remaining respiratory, stool, urine, and blood samples, will be frozen and stored at the study site laboratory. The trial provides a unique opportunity to investigate a number of TB biomarkers, which could discriminate active disease from TB infection as well as TB from non-TB pneumonia using transcriptomic approaches. It also allows future studies on *M.tb* molecular epidemiology, and further characterization of the proteomic, metabolic and immunologic profiles of children presenting with signs of severe pneumonia, with or without TB. Biological samples will be retained for 10 years after study completion, unless there are objections expressed by the adult participant and children’s parent(s)/guardian(s).

## Discussion

This study is designed to evaluate a new diagnostic test that aims to improve molecular confirmation of TB as well as provide a new monitoring tool for treatment response in children and PLHIV. To date, studies using stool as a target sample have been done with already existing molecular tests like Xpert and Xpert Ultra using stool, but few of those studies propose novel molecular platforms for validation or exclusively include the vulnerable populations of intended use (children or PLHIV with presumptive TB). Therefore, the relevance of this study relies not only on the use of stool as a sample for bacteriological confirmation, but also on the molecular non-Xpert based approach to it, as there is need for improvement molecular stool diagnostics. Further, the design is aligned with the latest WHO guidelines on diagnosis and management of TB in children and adolescents that, for the first time, include stool as a valid sample for TB testing. In fact, apart from the classical approach of using culture in a respiratory sample as a reference standard, we will assess the performance of this new diagnostic test against a robust composite reference standard that includes culture and Xpert Ultra in respiratory samples, Xpert Ultra in stool and LF-LAM in urine and against clinical diagnosis. In this way, we will both maximize the bacteriological confirmation and estimate the potential increase in bacteriological confirmation in those cases that start treatment in the absence of bacteriologic confirmation. It will also allow to see the contribution of each single TB test to bacteriological confirmation, including the expected additionality of que novel stool-based qPCR (all other test negative but qPCR positive).

Another relevant strength in the study design is the two week and two-month longitudinal follow-up to verify baseline disease classification in participants who do not initiate treatment. This will play a relevant role in both increasing the number of confirmed cases (after resampling if persistence of symptoms), but also in the reliability of the specificity estimations (only those who have tested negative at baseline, who did not start treatment and who do not present symptoms after 2 months will be classified as “unlikely TB”).

The study is designed to include those presumptive TB cases already detected by the routine procedures of the national TB programs or healthcare system workers and does not include those identified in community screening programs. Therefore, recruitment will rely on the performance of the already existing platforms for TB detection. While this is good practice and prevents the study from negatively impacting future clinical practice, the COVID-19 pandemic has impacted TB notifications in many study settings [1]. This may impact recruitment and the capacity to attain the expected sample size.

Though stool has already been recommended by the WHO as a sample for bacteriological confirmation in children, the implementation of stool-based diagnostics has not yet been widespread. Therefore, this study is highly relevant as it uses stool as the preferred sample. Also, the new qPCR platform has the potential to lower the limit of detection of Mtb in stool compared to currently available tests. If our project successfully demonstrates an increase in bacteriological confirmation, this platform has the potential to be adapted to a field-friendly point-of-care test that could be easily implemented in basic health care centers in Sub-Saharan Africa or resource-limited countries, in line with the priorities of the End TB strategy [38]. As a multi-national study with different epidemiological and environmental backgrounds, one can expect to face variability, but this will make results more robust and able to be applied in different environments.

## Data Availability

This is not applicable as it is a study protocol.
